# Anæsthetics, Ancient and Modern

**Published:** 1890-03

**Authors:** 


### Book Reviews.

### "ANÆSTHETICS, ANCIENT AND MODERN;"

BY DR. GEORGE FOY, F. R. C. S.: BAILLIERE, TINDALL & COX, LONDON; WEST, JOHNSON & CO., RICHMOND, VA.; 175 PAGES, 1889.

This is a very interesting resume of ancient and modern anæsthetics, their physiological action, therapeutic use, and mode of administration.

The historical part, dealing as it does with the gradual development of anæsthesia, from the period of crude drugs to the inception and discovery of modern anæthetics, is especially interesting, bringing up as it does in a brief summary the records of an almost forgotten past.

But aside from the interest, the book contains very valuable information. In discussing the advantages and disadvantages of the different anæsthetic agents, he has done so fully and without prejudice, although, as he says, he prefers chloroform.

The cases quoted of bad effects from anæsthesia, were selected to show that unpleasant results will occasionally occur, no matter what care is taken or skill is exercised.

The book is full of valuable statistics. Many of them I have never seen in print before. The writer in making quotations has given reference which greatly facilitates obtaining further information.

